# SLC7A7 is a prognostic biomarker correlated with immune infiltrates in non-small cell lung cancer

**DOI:** 10.1186/s12935-021-01781-7

**Published:** 2021-02-15

**Authors:** Wumin Dai, Jianguo Feng, Xiao Hu, Yongyi Chen, Qing Gu, Wangang Gong, Tingting Feng, Jie Wu

**Affiliations:** 1 Research center, Cancer Hospital of University of Chinese Academy of Sciences, Hangzhou, 310022 Zhejiang China; 2 Department of Abdominal Oncology, Cancer Hospital of University of Chinese Academy of Sciences, Hangzhou, 310022 Zhejiang China; 3 Clinical Laboratory, Cancer Hospital of University of Chinese Academy of Sciences, Hangzhou, 310022 Zhejiang China; 4 Department of Thoracic Oncology Radiotherapy, Cancer Hospital of University of Chinese Academy of Sciences, Hangzhou, 310022 Zhejiang China

**Keywords:** SLC7A7, Lymphocytes, Tumor-infiltrating, Prognosis, Non-small cell lung cancer

## Abstract

**Background:**

SLC7A7 (solute carrier family 7, amino acid transporter light chain, y + L system, member 7) is a critical gene in the regulation of cationic amino acid transport. However, the relationships between SLC7A7 and prognosis and tumor-infiltrating lymphocytes in different cancers remain unclear.

**Methods:**

SLC7A7 expression was analyzed using the Oncomine database and Tumor Immune Estimation Resource (TIMER) site. The enrichment of the GO (Gene Oncology) and KEGG (Kyoto Encyclopedia of Genes and Genomes) pathways was conducted by DAVID. We evaluated the influence of SLC7A7 on clinical prognosis using the PrognoScan database. The functional state of SLC7A7 in various types of cancers was analyzed by CancerSEA. The relationships between SLC7A7 and cancer immune infiltrates was investigated by TIMER. Furthermore, correlations between SLC7A7 expression and gene marker sets of immune infiltrates were analyzed by TIMER and Gene Expression Profiling Interactive Analysis (GEPIA). The expression of SLC7A7 was verified by GEO database and immunohistochemistry.

**Results:**

A lung cancer cohort study (GSE31210) showed that high SLC7A7 expression was associated with poor overall survival (OS) and relapse-free survival (RFS). In addition, SLC7A7 had a significant impact on the prognosis of diverse cancers. SLC7A7 expression was positively correlated with infiltrating levels of CD4 + and CD8 + T cells, macrophages, neutrophils and dendritic cells (DCs) in non-small cell lung cancer (NSCLC). SLC7A7 expression was also strongly correlated with various immune marker sets in NSCLC.

**Conclusions:**

These results indicated a role for SLC7A7 in infiltration of CD8 + T cells, CD4 + T cells, tumor-associated macrophages (TAMs), neutrophils and DCs in multiple cancers, and regulation of T cell exhaustion and Tregs in NSCLC. These findings suggest that SLC7A7 could be served as a biomarker for prognosis and immune infiltration in NSCLC.

## Background

Lung cancer (LC) is the most prevalent cancer and the leading cause of cancer-related mortality responsible for one in five cancer-related deaths worldwide [1]. Non-small cell lung cancer (NSCLC) comprises approximately 80–85% of all lung cancers diagnoses, divided by histology into squamous cell cancer and adenocarcinoma [2]. The 5-year overall survival rate for NSCLC is still less than 20% [3]. More recently, immunotherapy has been hailed as innovative therapeutic strategy for NSCLC. Nevertheless, there are certain serious challenges that limit the broader clinical application, such as limited clinical efficacy, the lack of effective predictive biomarkers and treatment-related adverse events (TRAEs) [4]. In addition, recent studies have reported that tumor-infiltrating lymphocytes, such as TAMs and tumor-infiltrating neutrophils (TINs), are closely associated with the prognosis and the efficacy of cancer immunotherapy [5, 6]. Therefore, it is urgent to define the immunophenotypes of tumor-immune interactions and identify novel immunotherapy targets in NSCLC.

Amino acid transporters have been classified into two families: SLC3 and SLC7. The SLC7A7 gene encodes the y^+^LAT1 light chain of system y^+^L, which binds to 4F2hc for transmembrane amino acid transport [7]. The transport system exports cationic amino acids such as arginine and lysine out of the cell across the membrane in both a Na^+^-independent and dependent manner [8, 9]. The role of SLC7A7 in lysinuric protein intolerance has been extensively studied. In addition, mutations in SLC7A7 causing cation transporter dysfunction are associated with a variety of clinical symptoms [10]. Overexpression of SLC7A7 is correlated with poor RFS and OS in patients with glioblastoma [11]. The expression profiles of SLC7A7, together with CSGAL-NACT1, were used to stratify multiple myeloma patients into two groups with different prognoses [12]. SLC7A7 was highly expressed in chemoresistant ovarian cancer and could modulate the influx/efflux of drugs from cells, and thus, regulate the chemotherapy response [13]. Moreover, downregulation of SLC7A7 was involved in acquired radioresistance [14]. More importantly, the mRNA expression of SLC7A7 was markedly increased in human monocytes during macrophage differentiation [15]. These findings point to the crucial role that SLC7A7 plays in cancer initiation and progression. However, the underlying functions and mechanisms of SLC7A7 in tumor progression and tumor immunology are still unclear.

## Methods

### Oncomine database analysis

Oncomine (https://www.oncomine.org/resource/login.html) is a publicly available tumor microarray database and data mining platform that includes 715 datasets, as well as 86,733 cancer and normal tissue samples. Gene expression analyses for a single gene or a set of genes can be performed across various types of cancer and include comparisons relative to normal controls, other cancer subtypes and various clinicopathological features [16]. In this study, the Oncomine database was employed to analyze the expression levels of the SLC7A7 gene in various cancers. The threshold was determined with the following criteria: P-value of 0.001 and fold change of 2, regardless of gene rank.

### Functional analysis of SLC7A7 and related genes

To identify enriched functional categories of SLC7A7 and related genes, the STRING (https://string-db.org/, version 11.0) was used to unveil the functional relationships for the top 100 genes that most relevant with SLC7A7 by constructing the PPI (protein–protein interaction) network using Cytoscape (version 8.2). Gene Oncology (GO) and KEGG pathway enrichment analysis was performed using the Database for Annotation, Visualization and Integrated Discovery (DAVID) (https://david.ncifcrf.gov/). Following the instructions of the DAVID manual, SLC7A7 and related genes were uploaded and the function charts were generated. The groups with a P-value < 0.05 and gene counts more than two were examined.

### PrognoScan database analysis

The correlation between SLC7A7 expression and survival in various cancer types was determined by Cox proportional hazards analysis using data from the PrognoScan database (http://www.abren.net/PrognoScan/; [17]. PrognoScan provides a comprehensive platform to explore the relationships between gene expression and patient prognosis across a large collection of publicly available cancer microarray datasets. The significance threshold was adjusted to a Cox P-value < 0.05.

### CancerSEA analysis

The functional state of SLC7A7 in various cancer types was analyzed by CancerSEA (http://biocc.hrbmu.edu.cn/CancerSEA/). CancerSEA is the first integrative database aimed at decoding different functional states of cancer cells at a single-cell resolution. CancerSEA depicts a cancer single-cell functional state atlas, covering 14 functional states (including stemness, invasion, metastasis, proliferation, EMT, angiogenesis, apoptosis, cell cycle, differentiation, DNA damage, DNA repair, hypoxia, inflammation and quiescence) of 41,900 cancer single cells from 25 cancer types [18]. Correlations between the gene of interest and functional state in different single-cell datasets were filtered by a correlation strength > 0.3 and a false discovery rate (FDR) (Benjamini & Hochberg) < 0.05.

### TIMER database analysis

Tumor Immune Estimation Resource (TIMER) is an integrative resource for investigating the molecular characterization of tumor-immune interactions across various cancer types (https://cistrome.shinyapps.io/timer/) [19]. TIMER utilizes a deconvolution statistical method to deduce the abundance of six tumor-infiltrating immune cells, including B cells, CD4 + T cells, CD8 + T cells, macrophages, neutrophils and DCs from The Cancer Genome Atlas (TCGA). We analyzed SLC7A7 expression in different types of cancer and the correlation of SLC7A7 expression with the abundance of immune infiltrates using the gene module. In addition, the correlation module was used to explore the relationships between SLC7A7 expression and gene markers of tumor infiltrates. These gene markers included markers of CD8 + T cells, T cells (general), B cells, monocytes, TAMs, M1 macrophages, M2 macrophages, neutrophils, natural killer (NK) cells, DCs, T-helper 1 (Th1) cells, T-helper 2 (Th2) cells, follicular helper T (Tfh) cells, T-helper 17 (Th17) cells, Tregs and exhausted T cells, which were referenced in prior studies [20–22]. The gene expression level was displayed with log2 RSEM.

### Gene correlation analysis in GEPIA

The comprehensive online database Gene Expression Profiling Interactive Analysis (GEPIA) (http://gepia.cancer-pku.cn/index.html) was used to further validate the significantly correlated genes in TIMER. GEPIA [23] is an interactive web analysis based on TCGA and the GTEx projects, which includes 9736 tumors and 8587 normal samples. Gene expression correlation analysis was performed for given sets of TCGA expression data. The correlation coefficient was determined by the Spearman method. SLC7A7 was used for the x-axis and other genes of interest were represented on the y-axis. Tumor and para-cancerous tissues of LUAD were used for analysis.

### Verification of SLC7A7 expression in GEO database

Gene transcript data of normal and tumor tissues was obtained from The National Center for Biotechnology Information (NCBI) The Gene Expression Omnibus (GEO). In this study, eight GEO series (GSE) were used (GSE19188, GSE19804, GSE31210, GSE32663, GSE43458, GSE44077, GSE75037 and GSE10072). The dataset was selected as follows: (I) the number of samples > 100 included normal and lung cancer tissues; (II) the number of samples in a single group of was > 20; (III) For GEO, the query (‘expression profiling by array’) AND (‘expression profiling by throughput sequencing’) AND ‘homo sapiens’ [organism] was used to return a list of all potential data sets to analyse. The GSE19188 dataset contains data from 45 lung adenocarcinoma tissue samples and 65 fresh frozen adjacent non-cancerous samples. The GSE19804 dataset consists of samples from 60 lung cancer patients and 60 normal controls. The GSE31210 dataset comprises samples from 20 normal lung and 226 primary lung cancer. The GSE32863 dataset used in this study consists of 58 LUAD and 58 adjacent non-tumor lung tissue. This GSE43458 dataset is comprised of 110 samples including 30 normal lung tissue and 80 lung adenocarcinoma tissue from never smoker. The GSE44077 dataset used comprises mRNA-array data from 65 normal lung samples and 56 lung cancer samples. The GSE75037 dataset is tabulated in a matrix consisting of tens of thousands of genes and 166 samples including 83 lung cancer and 83 non-malignant lung samples. The GSE10072 dataset including tissue from both normal lung tissues (N = 49) and lung cancer tissues (N = 58) amounts to 107 samples. The expression of SLC7A7 was analyzed using an unpaired t-test.

### Immunohistochemistry

For validation the expression of SLC7A7 in tumor and normal tissue, archival formalin-fixed paraffin-embedded (FFPE) specimens of 34 pairs of LUAD and normal tissue were obtained from the Zhejiang Cancer Hospital. All of the tumor samples were again confirmed by two independent pathologists in Clinical Pathology Department of the hospital. For immunohistochemistry, 5 μm thickness sections of LUAD and matched control were cut from FFPE biopsy specimens. Sections were placed on poly L-lysine coated glass slides and immunostained with ZSGB-BIO (PK-4001). Briefly, sections were deparaffinized by placing in a 60 °C oven for 30 min, followed by rehydration in graded ethanol. For antigen epitope retrieval, sections were immersed in sodium citrate buffer low pH. To quench endogenous peroxidase activity, the specimens were incubated with peroxidase-blocking solution for 15 min. The tissue section was treated with primary antibody for SLC7A7 (Abcam) at 1:100 dilution, followed by overnight incubation at 4 °C in a humidified chamber, followed by washes in 0.3% PBST, and incubation by secondary goat anti rabbit antibody for 2 h at room temperature. Staining was developed using a DAB (3,3´-Diaminobenzidine) chromogen kit and a light counterstaining with haematoxylin was used to reveal cells. The sections were dehydrated and cover-slipped with permanent media and observed independently by two pathologists for the unbiased scoring of the markers.

The scoring was categorized as weak, moderate and strong according to the semiquantitative immunoreactivity scoring system (IRS), which was determined by multiplying the percentage of positive area and the score of staining intensity. The score of staining intensity was defined by a four-tier grading system (0 = negative, 1 = weak, 2 = moderate and 3 = strong staining intensity). The scores for cell positivity were given in the range of 1 to 4, based on: 1 for positive staining in 0–25% of tumor cells, 2 for positive staining in 25–50% of tumor cells, 3 for positive staining in 51–75% of tumor cells and, 4 for positive staining in 75–100% of tumor cells. Further, for statistical analyses the IRS scores of 0–4 were treated as weak staining, scores of 5–8 as moderate staining and, scores of 9–12 as intense staining.

### Statistical analysis

Survival curves were generated by PrognoScan. The results generated in PrognoScan were performed with the hazard ratio (HR), 95% confidence interval (CI) and Cox P-value. The correlation of gene expression was evaluated by Spearman’s correlation and statistical significance, and the strength of the correlation was determined using the following criteria: 0.00–0.19, “very weak”; 0.20–0.39, “weak”; 0.40–0.59, “moderate”; 0.60–0.79, “strong”; 0.80–1.0, “very strong”. P-values < 0.05 were considered statistically significant. Statistical and graphical analyses were performed with GraphPad Prism, version 8.0 (GraphPad Software).

## Results

### Expression levels of SLC7A7 in different types of cancer

**Fig. 1:**
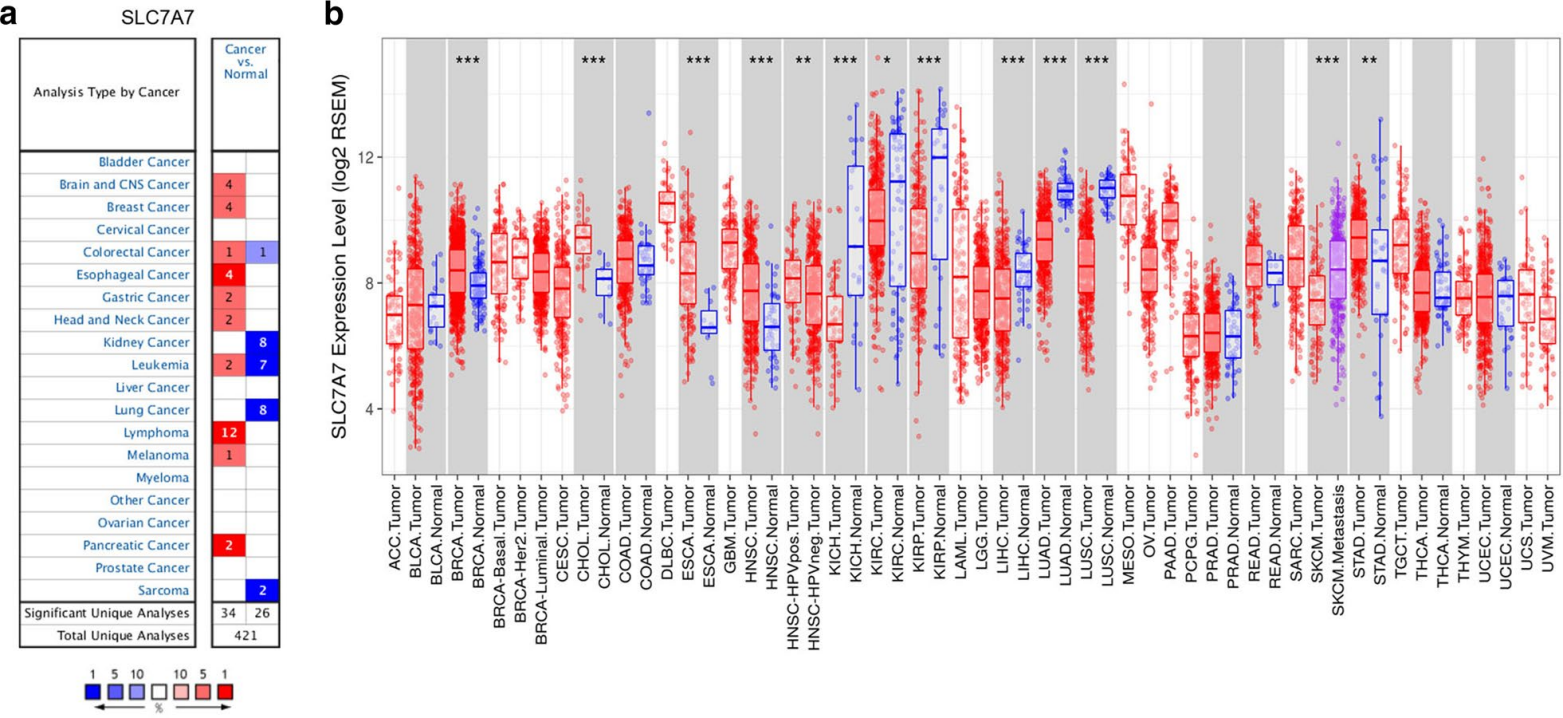
SLC7A7 expression levels in different types of human cancers. **a** Increased or decreased SLC7A7 in data sets of different cancers compared with normal tissues in the Oncomine database. **b** Human SLC7A7 expression levels in different tumor types from TCGA were determined by TIMER (*P < 0.05, **P < 0.01, ***P < 0.001)

First, the expression profiles of SLC7A7 in various cancer types and corresponding normal controls were investigated in the Oncomine database. We found that SLC7A7 expression was significantly higher in brain and CNS, breast, colorectal, esophageal, gastric, head and neck, leukemia, lymphoma, melanoma and pancreatic cancers, compared to the normal tissues (Fig. 1a). In addition, our results indicated that SLC7A7 mRNA expression levels in colorectal, kidney, leukemia, sarcoma and lung cancer were significantly underexpressed compared to the corresponding normal tissues in some data sets. The detailed results of SLC7A7 expression in various cancer types are summarized in Additional file 1: Table S1.

To further evaluate SLC7A7 expression in various cancer types, we investigated SLC7A7 expression using the RNA-seq data of multiple malignancies in TCGA. The results indicated that the expression levels of SLC7A7 were significantly increased in BRCA (breast invasive carcinoma), CHOL (cholangiocarcinoma), ESCA (esophageal carcinoma), HNSC (head and neck squamous cell carcinoma) and STAD (stomach adenocarcinoma), compared to adjacent control samples. However, SLC7A7 expression was significantly lower in KICH (kidney chromophobe), KIRC (kidney renal clear cell carcinoma), KIRP (kidney renal papillary cell carcinoma), LIHC (liver hepatocellular carcinoma), LUAD (lung adenocarcinoma) and LUSC (lung squamous cell carcinoma) than in normal controls (Fig. 1b). SLC7A7 showed the same expression trend in breast, esophageal, head and neck, kidney and lung cancers in both the microarray and RNA-seq data.

### Gene ontology (GO) classifications and KEGG mapping

**Fig. 2:**
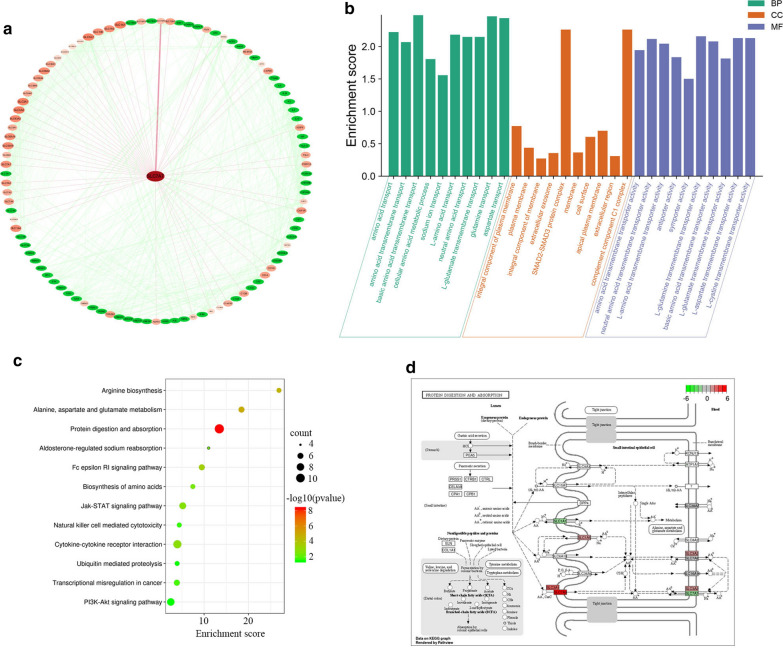
GO functional classification and KEGG pathway mapping of SLC7A7 and relevant genes. **a** The STRING database was used to analyze the top 100 relevant genes and Cytoscape was used to display the protein–protein interaction (PPI) network. **b** The distributions were summarized in three main categories: biological process, molecular function (MF), and cellular component (CC). The x-axis indicates different GO terms and the y-axis indicates the enrichment score in each category. **c** Scatter plot of enriched KEGG pathways statistics. The color and size of the dots represent the range of the p-value and the number of genes mapped to the indicated pathways, respectively. Top 10 enriched pathways are shown in the figure. **d** The map of “Protein digestion and absorption” was modified from the KEGG map. The red boxes indicated up-regulated genes and green boxes indicated down-regulated genes identified by KEGG mapping

The enrichment of the GO and KEGG pathways was conducted by web-based DAVID Bioinformatics Resources 6.8. SLC7A7 and the related genes were assigned to 117 GO terms, including 39 biological processes, 24 cellular components and 54 molecular function terms. The most significantly enriched GO-term in the molecular function was “amino acid transmembrane transporter activity”. GO cellular compartment analysis showed that SLC7A7 and related genes were highly enriched in integral component of plasma membrane. The most prevalent biological processes assignment was “amino acid transport” (Fig. 2a, b).

KEGG database is a collection of various pathways, representing the molecular interactions and reaction networks. To identify signalling pathways involved in SLC7A7, we had mapped the KEGG database and found that the relevant genes were significantly enriched in 28 KEGG pathways. The relevant genes were highly clustered in several signalling pathways, such as “protein digestion and absorption”, “alanine, aspartate and glutamate metabolism”, “arginine biosynthesis” and “Fc epsilon RI signaling pathway” (Fig. 2c, d).

### Prognostic potential of SLC7A7 in cancer

**Fig. 3:**
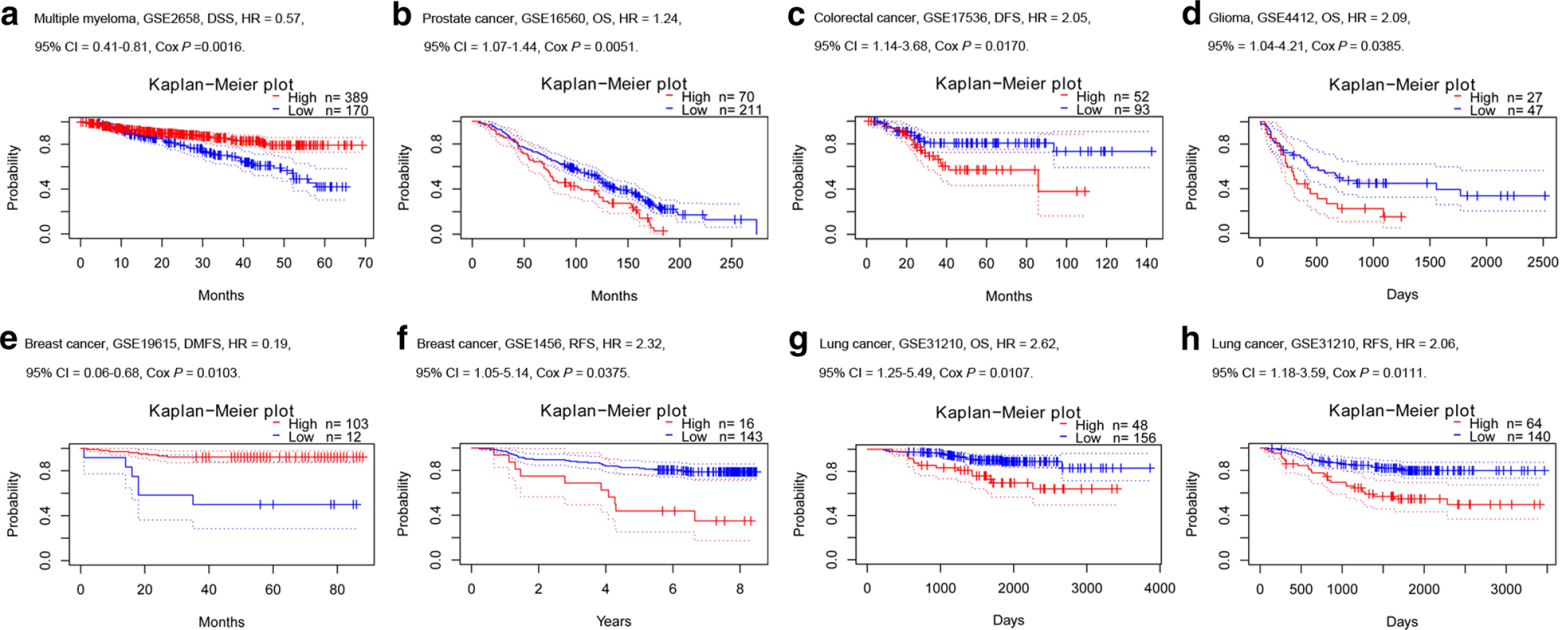
Kaplan–Meier survival curves comparing the high and low expression of SLC7A7 in different types of cancer in PrognoScan (**a**–**h**). **a** Survival curves for disease-specific survival (DSS) in one multiple myeloma cohort (GSE2658, n = 559). **b**–**d** High SLC7A7 expression was correlated with poor OS in the prostate cancer cohort (GSE16560, n = 281), poor disease-free survival (DFS) in the colorectal cancer cohort (GSE17536, n = 145) and poor OS in the glioma cohort (GSE4412, n = 74). **e** High SLC7A7 expression was correlated with better DMFS in one breast cancer cohort (GSE19615, n = 115). **f** High SLC7A7 expression was correlated with better RFS in one breast cancer cohort (GSE1456, n = 159). **g**, **h** High SLC7A7 expression was correlated with poor OS and RFS in the lung cancer cohort (GSE31210, n = 204)

We investigated the correlations between the mRNA expression levels of SLC7A7 and prognosis in cancer patients using the PrognoScan database. Notably, the analysis revealed that SLC7A7 expression was significantly correlated with prognosis in six types of cancer, including multiple myeloma, prostate, colorectal, glioma, breast and lung cancer (Fig. 3a–h). Interestingly, one cohort (GSE19615) including 115 samples showed that high SLC7A7 expression was associated with a better prognosis in breast cancer (distant metastasis-free survival [DMFS]; HR = 0.19, 95% CI 0.06–0.68, P = 0.0103). Another cohort (GSE1456) including 159 samples showed that SLC7A7 expression was associated with a worse prognosis in breast cancer (relapse-free survival [RFS]; HR = 2.32, 95% CI 1.05–5.14, P = 0.0375). However, the poor prognosis for lung cancer (OS: HR = 2.62, 95% CI 1.25–5.49, P = 0.0107; RFS: HR = 2.06, 95% CI 1.18–3.59, P = 0.0111) was correlated with higher SLC7A7 expression (Fig. 3g, h). Hence, these results confirmed that SLC7A7 is of great importance for assessing the prognosis of certain types of cancer, with increased and reduced SLC7A7 expression having different prognostic value depending on the type of cancer.

### Functional state of SLC7A7 across different cancer types

**Fig. 4:**
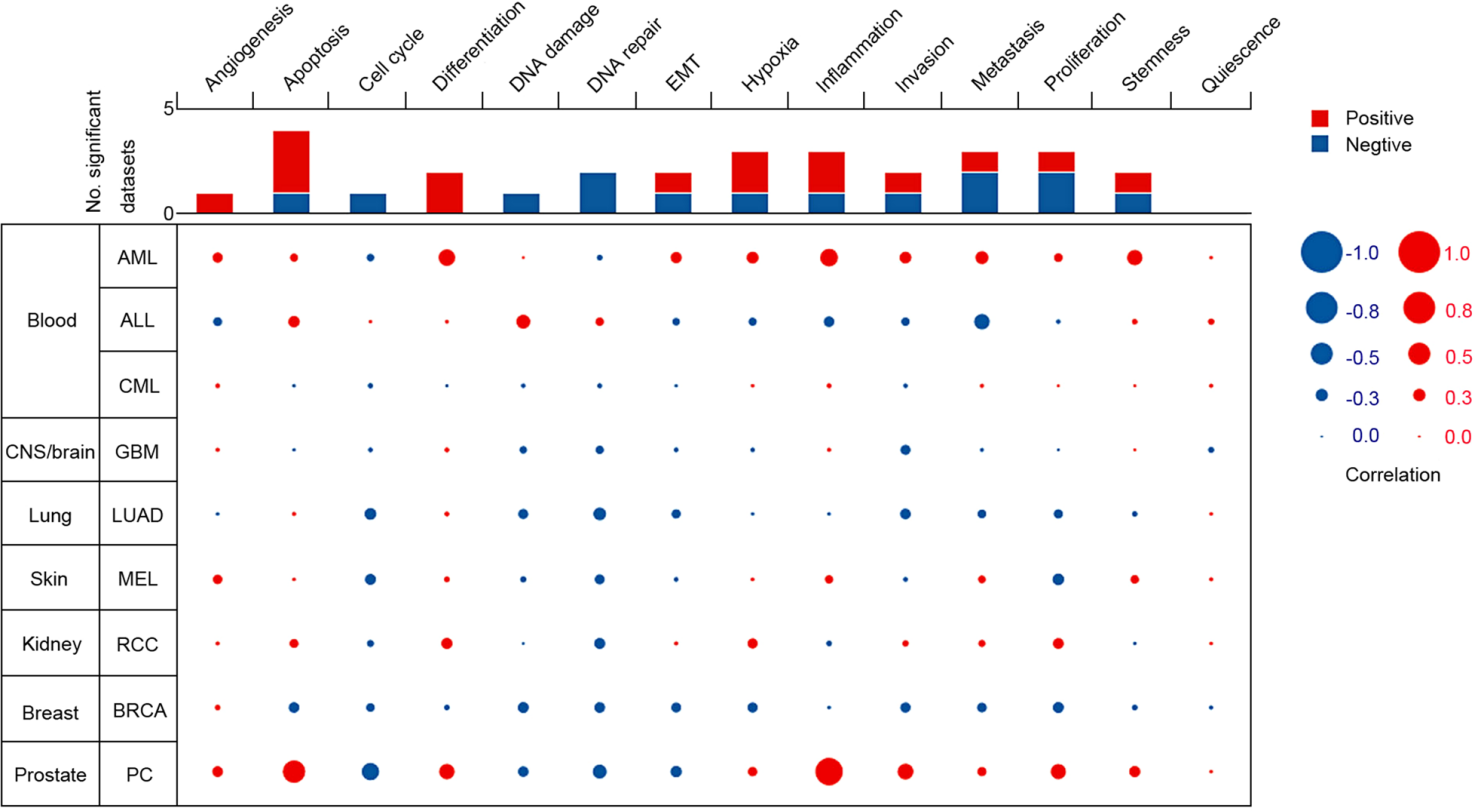
The functional state of SLC7A7 across nine types of cancer. The red plots indicated that SLC7A7 was positively correlated with the functional state while the blue plots indicated that SLC7A7 was negatively correlated with the functional state identified by CancerSEA

To better understand the relevance and underlying mechanisms of SLC7A7 expression in cancer, we investigated the functional state of SLC7A7 across different cancer types in the CancerSEA database. SLC7A7 has been investigated at the single-cell level in nine types of cancer (Fig. 4), including AML (acute myeloid leukemia), ALL (acute lymphoblastic leukemia), CML (chronic myelogenous leukemia), GBM (glioblastoma), LUAD, MEL (melanoma), RCC (renal cell carcinoma), BRCA and PC (prostate cancer). SLC7A7 was positively correlated with apoptosis (ρ = 0.509, *P* = 0.028) and inflammation (ρ = 0.645, *P* = 0.004) in PC. SLC7A7 was negatively correlated with metastasis (ρ =  − 0.334, *P* = 0.023) in ALL. SLC7A7 was positively correlated with differentiation (ρ = 0.36, *P* < 0.001), inflammation (ρ = 0.39, *P* < 0.001) and quiescence (ρ = 0.33, *P* < 0.001) in AML. However, SLC7A7 was not significantly correlated with any of the 14 functional states in NSCLC.

### Correlation between SLC7A7 expression and immune infiltration levels in lung cancer

**Fig. 5:**
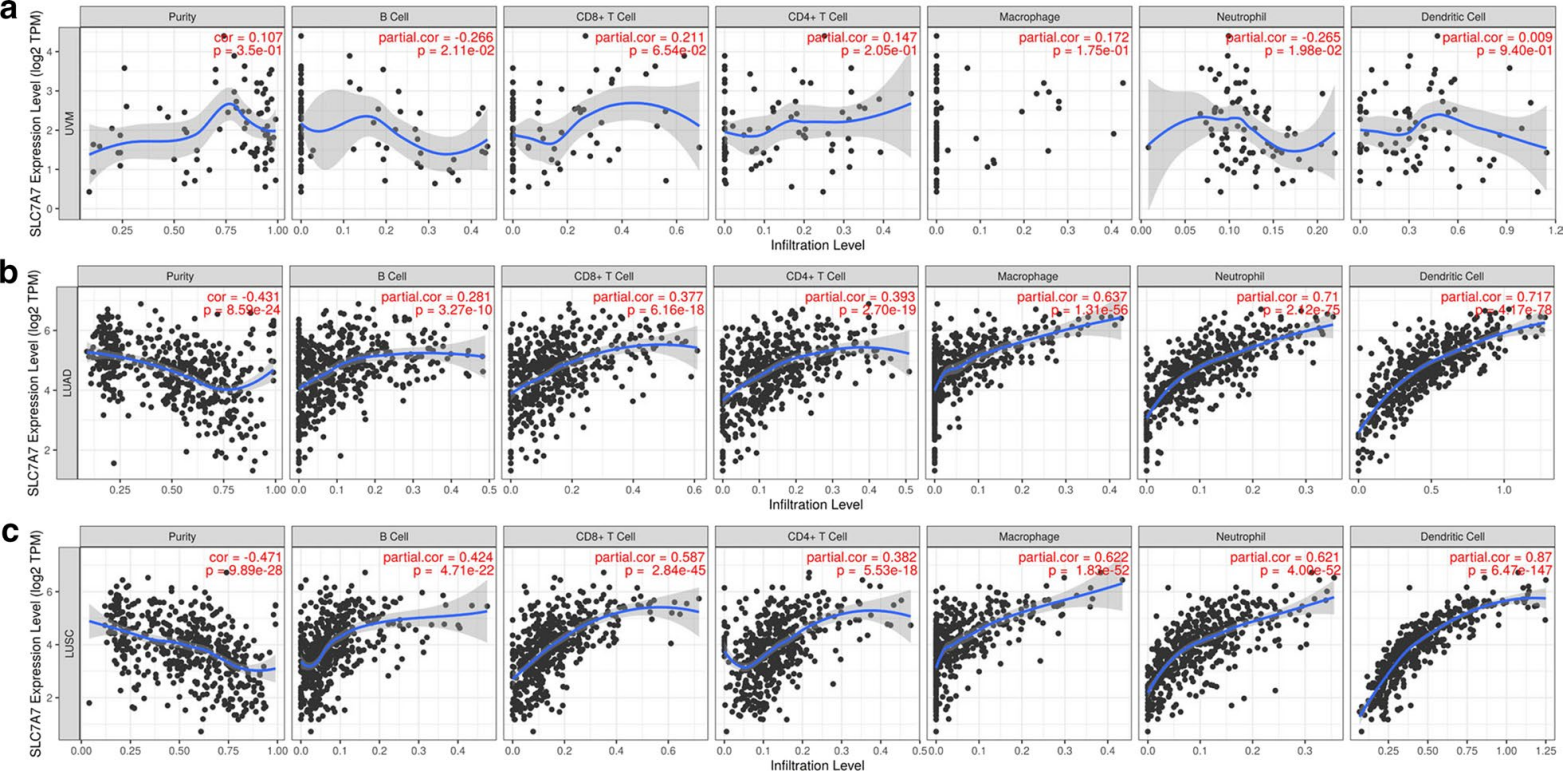
Correlation of SLC7A7 expression with immune infiltration levels in KIRC, LUAD and LUSC. **a** SLC7A7 expression displayed no significant correlations with tumor purity and infiltrating levels of CD8 + T cells, CD4 + T cells or neutrophils in KIRC. SLC7A7 expression showed a very weak correlation with B cells, macrophages and DCs in KIRC. **b** SLC7A7 expression was negatively related to tumor purity and showed significant positive correlations with infiltrating levels of B cells, CD8 + T cells, CD4 + T cells, macrophages, neutrophils and DCs in LUAD. **c** SLC7A7 expression was negatively related to tumor purity and displayed significant positive correlations with infiltrating levels of B cells, CD8 + T cells, CD4 + T cells, macrophages, neutrophils and DCs in LUSC

Compelling evidence has demonstrated that tumor-infiltrating lymphocytes are significantly associated with survival in cancer. Therefore, we investigated whether SLC7A7 expression was related to immune infiltration levels in lung cancer by TIMER. Tumor purity is an important factor affecting the analysis of immune infiltration. Interestingly, our results indicated that SLC7A7 expression was correlated with poor prognosis and high immune infiltration in NSCLC. SLC7A7 was highly expressed in monocytes (non-classical and classical) and B cells (naïve; Additional file 2: Figure S1). In contrast, SLC7A7 expression was not significantly correlated with tumor purity or infiltrating levels of CD8 + T cells, CD4 + T cells or neutrophils in UVM (Fig. 5a). SLC7A7 expression levels were positively correlated with infiltrating levels of B cells (r = 0.281, P = 3.27E−10), CD8 + T cells (r = 0.377, P = 6.16E−18), CD4 + T cells (r = 0.393, P = 2.7E−19), macrophages (r = 0.637, P = 1.31E−56), neutrophils (r = 0.71, P = 2.12E−75) and DCs (r = 0.717, P = 4.17E−78) in LUAD (Fig. 5b). SLC7A7 expression levels were also positively correlated with infiltrating levels of B cells (r = 0.424, P = 4.71E−22), CD8^+^ T cells (r = 0.587, P = 2.84E−45), CD4^+^ T cells (r = 0.282, P = 5.53E−18), macrophages (r = 0.622, P = 1.83E−52), neutrophils (r = 0.621, P = 4.00E−52) and DCs (r = 0.87, P = 6.47E−147) in LUSC (Fig. 5c). These findings strongly suggest that SLC7A7 plays an important role in immune infiltration in NSCLC, especially infiltration of neutrophils, macrophages and DCs.

### Correlation analysis between SLC7A7 expression and immune marker sets

To investigate the relationship between SLC7A7 and the diverse immune infiltrating cells, we analyzed the correlations between SLC7A7 expression and immune marker genes of different immune cells, including CD8 + T cells, T cells (general), B cells, monocytes, TAMs, M1 and M2 macrophages, neutrophils, NK cells and DCs in NSCLC (Table 1). We also examine the different functional phenotypes of T cells, including Th1 cells, Th2 cells, Tfh cells, Th17 cells and Tregs, as well as exhausted T cells. After the correlations were adjusted for purity, the results indicated that SLC7A7 was significantly associated with most immune marker sets of various immune cells and different T cells in NSCLC.

**Table 1:** Correlation analysis between SLC7A7 and relate genes and markers of immune cells in TIMER

Description	Gene markers	LUAD				LUSC			
		None		Purity		None		Purity	
		Cor	P	Cor	P	Cor	P	Cor	P
CD8 + T cell	CD8A	0.52	***	0.402	***	0.723	***	0.683	***
	CD8B	0.444	***	0.355	***	0.55	***	0.538	***
T cell (general)	CD3D	0.545	***	0.416	***	0.765	***	0.699	***
	CD3E	0.569	***	0.437	***	0.797	***	0.734	***
	CD2	0.604	***	0.489	***	0.812	***	0.759	**
B cell	CD19	0.313	***	0.13	**	0.539	***	0.389	***
	CD79A	0.299	***	0.125	**	0.586	***	0.449	***
Monocyte	CD86	0.793	***	0.743	***	0.897	***	0.863	***
	CSF1R	0.725	***	0.67	***	0.897	***	0.86	***
TAM	CCL2	0.455	***	0.371	***	0.634	***	0.57	***
	CD68	0.797	***	0.758	***	0.795	***	0.75	***
	IL10	0.619	***	0.53	***	0.714	***	0.672	***
M1 Macrophage	NOS2	0.078	0.0817	0.157	***	− 0.013	0.767	0.004	0.926
	IRF5	0.441	***	0.514	***	0.156	**	0.111	*
	PTGS2	− 0.15	***	− 0.13	**	0.044	0.324	− 0.055	0.23
M2 Macrophage	CD163	0.654	***	0.713	***	0.865	***	0.834	***
	VSIG4	0.732	***	0.763	***	0.883	***	0.863	***
	MS4A4A	0.733	***	0.777	***	0.898	***	0.876	***
Neutrophils	CEACAM8	0.266	***	0.226	***	0.129	**	0.113	*
	ITGAM	0.66	***	0.71	***	0.779	***	0.71	***
	CCR7	0.29	***	0.45	***	0.659	***	0.556	***
	KIR2DL1	0.105	*	0.156	**	0.306	**	0.268	***
	KIR2DL3	0.185	***	0.279	***	0.367	**	0.327	***
	KIR2DL4	0.204	***	0.281	***	0.403	***	0.345	***
	KIR3DL1	0.151	***	0.213	***	0.467	***	0.432	***
	KIR3DL2	0.163	***	0.251	***	0.455	***	0.402	***
	KIR3DL3	0.049	0.274	0.075	0.091	0.143	**	0.139	**
	KIR2DS4	0.088	0.050	0.17	***	0.338	***	0.317	**
Dendritic cell	HLA-DPB1	0.602	***	0.521	***	0.893	***	0.858	***
	HLA-DQB1	0.41	***	0.306	***	0.703	***	0.641	***
	HLA-DRA	0.628	***	0.553	***	0.905	***	0.88	***
	HLA-DPA1	0.612	***	0.539	***	0.904	***	0.878	***
	CD1C	0.32	***	0.236	***	0.54	***	0.37	***
	NRP1	0.175	***	0.142	**	0.581	***	0.494	***
	ITGAX	0.694	***	0.615	***	0.767	***	0.677	***
Th1	TBX21	0.5	***	0.368	***	0.695	***	0.615	***
	STAT4	0.455	***	0.325	***	0.704	***	0.612	***
	STAT1	0.465	***	0.377	***	0.529	***	0.493	***
	IFN-γ (IFNG)	0.45	***	0.344	***	0.524	***	0.489	***
	TNF-α (TNF)	0.396	***	0.269	***	0.363	***	0.221	***
Th2	GATA3	0.313	***	0.438	***	0.345	***	0.251	***
	STAT6	0.031	0.488	0.02	0.645	− 0.025	0.638	− 0.055	0.228
	STAT5A	0.564	***	0.65	***	0.645	***	0.549	***
	IL13	0.094	*	0.177	***	0.327	***	0.269	***
Tfh	BCL6	− 0.141	**	− 0.115	**	− 0.177	***	− 0.15	***
	IL21	0.207	***	0.276	***	0.421	***	0.362	***
Th17	STAT3	− 0.095	*	− 0.092	*	0.162	***	0.089	0.053
	IL17A	0.193	***	0.267	***	0.243	**	0.192	***
Treg	FOXP3	0.421	***	0.537	***	0.742	***	0.654	***
	CCR8	0.458	***	0.578	***	0.753	***	0.687	***
	STAT5B	0.216	***	0.235	***	0.133	**	0.134	**
	TGFβ (TGFB1)	0.304	***	0.394	***	0.215	***	0.076	0.097
T cell exhaustion	PD-1 (PDCD1)	0.356	***	0.485	***	0.69	***	0.612	***
	CTLA4	0.394	***	0.531	***	0.734	***	0.649	***
	LAG3	0.326	***	0.434	***	0.617	***	0.561	***
	TIM-3 (HAVCR2)	0.787	***	0.828	***	0.95	***	0.936	***
	GZMB	0.339	***	0.454	***	0.639	***	0.562	***

*p* < 0.05 were considered to be statistically significant (*p < 0.05, **p < 0.01, ***p < 0.001)

**Fig. 6:**
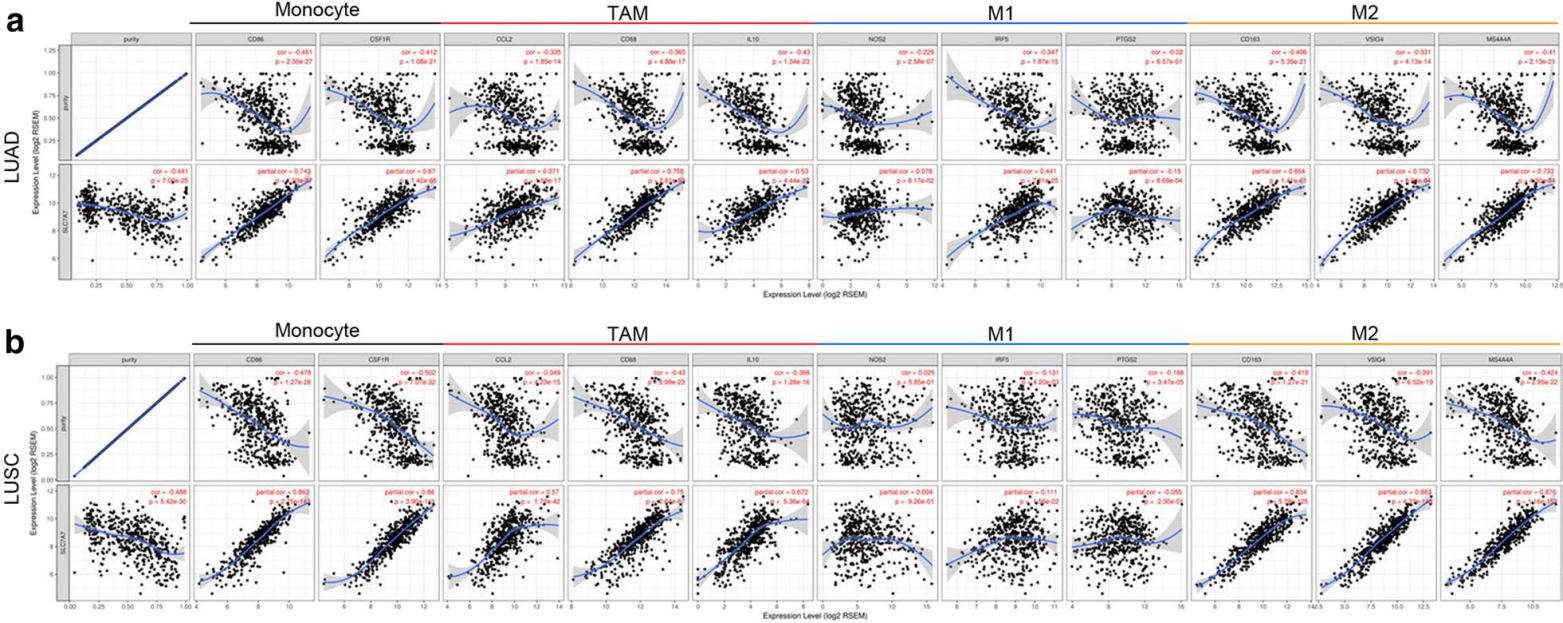
SLC7A7 expression correlated with macrophage polarization in NSCLC. Markers included CD86 and CSF1R of monocytes; CCL2, CD68 and IL10 of TAMs; NOS2, IRF5 and PTGS2 of M1 macrophages; and CD163, VSIG4 and MS4A4A of M2 macrophages. **a** Scatterplots of correlations between SLC7A7 expression and gene markers of monocytes, TAMs and M1 and M2 macrophages in LUAD. **b** Scatterplots of correlations between SLC7A7 expression and gene markers of monocytes, TAMs and M1 and M2 macrophages in LUSC

Intriguingly, we discovered that the expression levels of most marker set of monocytes, TAMs and M2 macrophages were highly correlated with SLC7A7 expression in NSCLC (Table 1). Specifically, we found that expression of SLC7A7 was significantly correlated with chemokine (C–C motif) ligand (CCL)-2, CD68, IL10 of TAMs, IRF5 of M1 phenotype, CD163, VSIG4 and MS4A4A of M2 phenotype in NSCLC (P < 0.0001; Fig. 6a, b). Furthermore, we validated the relationship between SLC7A7 expression and the above markers of monocytes and TAMs in GEPIA. The results showed that correlations between SLC7A7 and markers of monocytes and TAMs were highly analogous to those in TIMER (Table 2 and Additional file 3: Figure S2). These findings suggest that SLC7A7 may regulate macrophage polarization in NSCLC. DC markers such as HLA-DPB1, HLA-DRA, HLA-DPA1 and ITGAX also showed significant correlations with SLC7A7 expression, which indicated that there is a strong relationship between SLC7A7 and DC infiltration. Moreover, for Treg cells, SLC7A7 was positively correlated with FOXP3 and CCR8 in NSCLC. DCs can promote tumor metastasis by increasing Treg cells and attenuating CD8 + T cell cytotoxicity [24]. More attention should therefore be focused on SLC7A7 as a key factor mediating DC infiltration and tumor metastasis.

**Table 2:** Correlation analysis between SLC7A7 and relate genes and markers of monocyte and macrophages in GEPIA

Description	Gene markers	LUAD				LUSC			
		Tumor		Normal		Tumor		Normal	
		R	*P*	R	*P*	R	*P*	R	*P*
Monocyte	CD86	0.77	***	0.43	***	0.87	***	0.51	***
	CD115 (CSF1R)	0.73	***	0.41	**	0.89	***	0.51	***
TAM	CCL2	0.47	***	− 0.21	0.11	0.63	***	− 0.32	*
	CD68	0.77	***	0.56	***	0.75	***	0.67	***
	IL10	0.63	***	0.012	0.93	0.69	***	− 0.12	0.4
M1 Macrophage	INOS (NOS2)	0.24	***	0.0093	0.94	−7E−04	0.99	0.12	0.39
	IRF5	0.51	***	0.56	***	0.12	**	0.51	**
	COX2 (PTGS2)	− 0.081	0.074	− 0.18	0.18	0.053	0.25	− 0.14	0.34
M2 Macrophage	CD163	0.71	***	0.44	***	0.86	***	0.43	**
	VSIG4	0.76	***	0.65	***	0.86	***	0.74	***
	MS4A4A	0.76	***	0.55	***	0.87	***	0.52	***

*p* < 0.05 were considered to be statistically significant (*p < 0.05, **p < 0.01, ***p < 0.001)

We were excited to observe a strong positive correlation between SLC7A7 and marker genes of T cell exhaustion, especially TIM-3 (Table 2), which suggested that high SLC7A7 expression plays a crucial role in TIM-3-mediated T cell exhaustion. Therefore, these results further confirm our findings that SLC7A7 is specifically correlated with immune infiltrating cells in NSCLC, indicating that SLC7A7 plays a vital role in immune escape in the lung cancer microenvironment.

### SLC7A7 expression verification

**Fig. 7:**
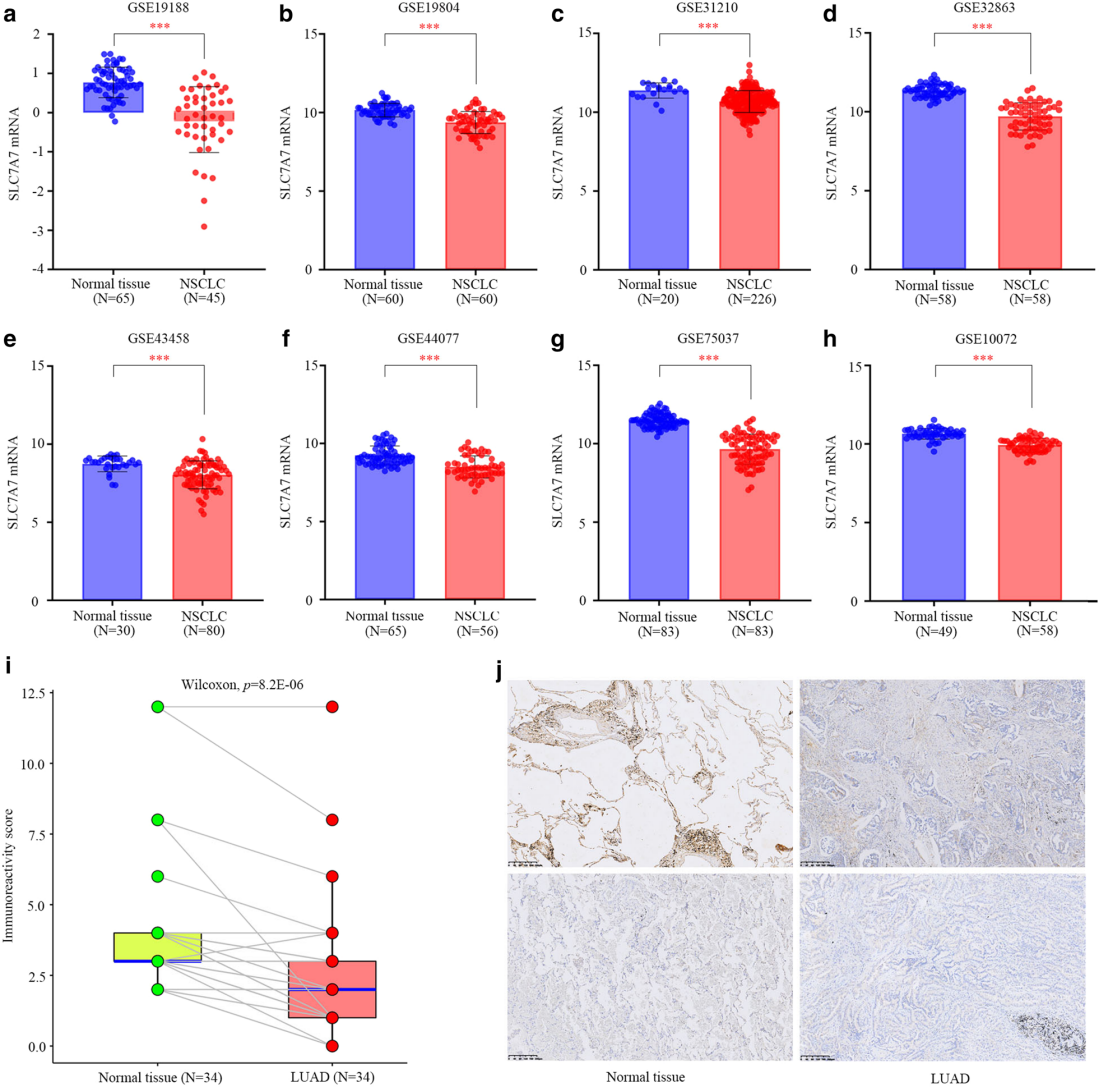
The expression of SLC7A7 in NSCLC and normal tissues was verified by GEO database and immunohistochemistry. **a**–**h** The expression of SLC7A7 was significantly lower in 666 cases of NSCLC than 479 controls in the eight datasets (GSE19188, GSE19804, GSE31210, GSE32663, GSE43458, GSE44077, GSE75037 and GSE10072). The expression of SLC7A7 was analyzed using an unpaired t-test. **i**–**j** SLC7A7 immunohistochemistry in lung adenocarcinoma tissues and adjacent normal lung tissues. The expression score of SLC7A7 was analyzed using wilcoxon

There were eight datasets that met the criteria, as follows: GSE19188, GSE19804, GSE31210, GSE32663, GSE43458, GSE44077, GSE75037 and GSE10072. There were 666 cases of NSCLC and 479 normal controls. The results of a nonpaired t-test showed that the expression of SLC7A7 was significantly lower in 666 cases of NSCLC than 479 cases in the control group (Fig. 7a–h). We then analyzed 34 pairs of LUAD tissues and the corresponding normal lung tissues to verify SLC7A7 expression. IHC staining showed that SLC7A7 was significantly down-regulated in LUAD (Fig. 7i, j). In general, like the TCGA database based on RNA high-throughput sequencing, SLC7A7 was also poorly expressed in the microarray datasets in NSCLC tissues, which can be used as a marker of LUAD prognosis and immune cell infiltration.

## Discussion

SLC7A7 partners with SLC3A2/4F2hc to mediate the transport of cationic amino acid. More importantly, it is well known that SLC7A7 is indispensable for the transport of L-arginine in monocytes [25]. However, downregulation of SLC7A7 in vitro induces an inflammatory phenotype of human macrophages and airway epithelial cells in an arginine content independent manner. These results suggest that the SLC7A7/y + LAT1 transporter has a still unknown immunomodulatory function, which is independent of arginine availability [26]. Herein, variations in SLC7A7 expression levels were found to be correlated with prognosis in different types of cancer. Elevated SLC7A7 expression was a predictor for poor prognosis in NSCLC. Interestingly, single-cell analyses showed that SLC7A7 was of no significance to the functional states in NSCLC. Furthermore, our results indicated that the expression levels of SLC7A7 were associated with immune infiltration levels and diverse immune marker sets. Hence, our study provides novel insights for understanding the potential role of SLC7A7 in tumor immunology and its use as a cancer biomarker or therapy target.

In the present study, we examined SLC7A7 expression and prognostic values in various types of cancer using independent datasets in Oncomine and GEO data in PrognoScan. SLC7A7 was differentially expressed between cancer and normal tissues in many types of cancer. Compared with normal tissues, SLC7A7 was overexpressed in brain and CNS, breast, colorectal, esophageal, gastric, head and neck, leukemia, lymphoma, melanoma and pancreatic cancers, while some data sets showed that SLC7A7 was underexpressed in colorectal, kidney, leukemia, sarcoma and lung cancers based on Oncomine (Fig. 1a). However, the expression of SLC7A7 was higher in BRCA, CHOL, ESCA, HNSC and STAD tissues than in corresponding normal tissues, but was lower in KICH, KIRC, KIRP, LIHC, LUAD and LUSC cancers by analysis of TCGA data (Fig. 1b). The discrepancies in SLC7A7 expression levels in different cancer types in different databases may be a reflection of data collection methods and the underlying mechanisms pertinent to different biological properties. Nevertheless, in these databases we found consistent SLC7A7 expression in breast, esophageal, head and neck, kidney and lung cancer. Analysis of data from PrognoScan revealed that increased SLC7A7 expression correlated with poor prognosis in several tumor types (prostate, colorectal, glioma, breast and lung cancer). Multiple myeloma and breast cancer were exceptions where high SLC7A7 expression showed a better prognosis. In one dataset of PrognoScan, high SLC7A7 expression could be used as an independent risk factor for poor prognosis in NSCLC (Fig. 2g, h). To better understand the relevance and underlying mechanisms of SLC7A7 expression in cancer, we investigated the functional states of SLC7A7 across different cancer types. SLC7A7 was not significantly correlated with any of the 14 functional states in NSCLC.

Another important aspect of this study was the correlation between SLC7A7 expression and diverse immune infiltration levels in cancer, especially in NSCLC. Our results demonstrated that SLC7A7 expression was moderately to strongly correlated with infiltration levels of macrophages, neutrophils and DCs, and was significantly correlated with infiltration levels of CD8 + and CD4 + T cells and B cells in NSCLC (Fig. 4b, c). In addition, the correlation between SLC7A7 expression and marker genes of immune cells suggested a role for SLC7A7 in regulating tumor immunology in NSCLC. First, there was no correlation or a negative correlation between gene markers of M1 macrophages and SLC7A7 expression, whereas SLC7A7 expression was moderately to strongly correlated with M2 macrophage markers such as CD163, VSIG4 and MS4A4A (Table 1, 2). These results reveal the potential regulatory role of SLC7A7 in TAM polarization. Moreover, our results indicated that SLC7A7 has the potential to activate Tregs and induce T cell exhaustion. Increased SLC7A7 expression was positively correlated with the expression of Treg and T cell exhaustion markers FOXP3, CCR8, STAT5B, TGFB1, TIM-3, PD-1, CTLA4 and LAG3 in NSCLC. TIM-3, an important surface protein on exhausted T cells [27], was highly correlated with SLC7A7 expression in NSCLC. Furthermore, SLC7A7 expression was significantly correlated with the regulation of several markers of T helper cells (Th1, Th2, Tfh and Th17) in NSCLC. These correlations suggest a potential mechanism by which SLC7A7 regulates T cell functions in NSCLC. Thus, SLC7A7 plays an important role in the recruitment and regulation of immune infiltrating cells in NSCLC.

Recent studies suggest an underlying mechanism to plausibly explain why SLC7A7 expression is associated with immune infiltration and poor prognosis. One the one hand, the relative deficiency of intracellular arginine due to the elevation of SLC7A7 level in tumor cells could promote cell migration and invasion [28], and inhibit cell apoptosis [29]. One the other hand, overexpression of SLC7A7 in tumor cells in turn leads to elevated arginine in the microenvironment. There are mainly two cross-inhibitory interactions metabolic pathways of arginine in macrophages: it is converted into NO and citrulline via inducible nitric oxide synthase (iNOS) in M1 macrophages or metabolized by arginase for production of ornithine and urea in M2 macrophages [30–32]. M2/arginase macrophages may be more effective for inducing extracellular arginine deprivation due to lack of intracellular reconstitutive mechanism for arginine recycling [33]. Moreover, M2 type macrophages increase their arginine transport capacity via endogenous arginase-mediated synthesis of polyamines, which can then further expand the arginase-based metabolism in a positive feed-back loop [34]. The microenvironment deprivation of L-arginine in turn leads to the suppression of T-cell activation, proliferation, differentiation and function [35].

In summary, elevated SLC7A7 expression is correlated with poor prognosis and enhanced infiltration of macrophages, neutrophils and DCs in multiple cancers, especially in NSCLC. In addition, SLC7A7 expression may be involved in the regulation of TAMs, DCs, T cell exhaustion and Tregs in NSCLC. Therefore, SLC7A7 may play a prominent role in immune cell infiltration and serve as a prognosis biomarker for NSCLC.

## Supplementary Information

**Additional file 1.** The expression of SLC7A7 in various cancer types.

**Additional file 2.** Boxplot of expression of SLC7A7 across diverse immune cells.

**Additional file 3.** The correlations between SLC7A7 and makers of monocytes and TAMs were validated by GEPIA.

## Data Availability

The datasets used and/or analyzed during the current study are available from the corresponding author on reasonable request.

## Abbreviations

TIMER: Tumor immune estimation resource
OS: Overall survival
RFS: Relapse-free survival
DC: Dendritic cells
NSCLC: Non-small cell lung cancer
TAMs: Tumor-associated macrophages
TINs: Tumor-infiltrating neutrophils
NK: Natural killer cell
Th1: T-helper 1 cell
Th2: T-helper 2 cell
Tfh: Follicular helper T cell
Th17: T-helper 17 cell
GEPIA: Gene expression profiling interactive analysis
HR: Hazard ratio
CI: Confidence interval
DMFS: Distant metastasis-free survival
BRCA: Breast invasive carcinoma
CHOL: Cholangiocarcinoma
ESCA: Esophageal carcinoma
HNSC: Head and neck squamous cell carcinoma
STAD: Stomach adenocarcinoma
KICH: Kidney chromophobe
KIRC: Kidney renal clear cell carcinoma
KIRP: Kidney renal papillary cell carcinoma
LIHC: Liver hepatocellular carcinoma
LUSC: Lung squamous cell carcinoma
AML: Acute myeloid leukemia
ALL: Acute lymphoblastic leukemia
CML: Chronic myelogenous leukemia
GBM: Glioblastoma
MEL: Melanoma
RCC: Renal cell carcinoma
PC: Prostate cancer
