# Supplementary material for: SLC7A7 is a prognostic biomarker correlated with immune infiltrates in non-small cell lung cancer
Source: Cancer Cell Int. 2021 Feb 15;21:106. doi: 10.1186/s12935-021-01781-7 (PMC7905560; doi:10.1186/s12935-021-01781-7)
Supplement: Supplementary file 1 — Additional file 1. The expression of SLC7A7 in various cancer types. [file 12935_2021_1781_MOESM1_ESM.docx]

Additional file 1

| Cancer | Cancer type | P-Value | FC | Rank (%) | Sample | Reference |
| --- | --- | --- | --- | --- | --- | --- |
| Brain and CNS Cancer | Glioblastoma | 1.19E-10 | 4.068 | 3% | 542 | No |
|  | Glioblastoma | 8.63E-6 | 5.469 | 3% | 22 | 16697959 |
|  | Glioblastoma | 4.20E-6 | 3.489 | 5% | 27 | 16204036 |
|  | Glioblastoma | 1.35E-8 | 2.062 | 10% | 81 | 16616334 |
| Breast cancer | Invasive Ductal Breast Carcinoma Stroma | 4.24E-4 | 2.838 | 3% | 7 | 17914389 |
|  | Medullary Breast Carcinoma | 2.99E-10 | 2.885 | 5% | 32 | 22522925 |
|  | Breast Carcinoma | 4.11E-4 | 2.376 | 10% | 14 | 22522925 |
|  | Invasive Breast Carcinoma Stroma | 2.71E-12 | 10.897 | 9% | 53 | 18438415 |
| Colorectal cancer | Colon Carcinoma Epithelia | 6.50E-9 | 2.798 | 2% | 5 | 20957034 |
|  | Colon Adenoma | 8.43E-5 | -2.458 | 9% | 5 | 20957034 |
| Esophageal cancer | Esophageal Adenocarcinoma | 3.85E-20 | 4.985 | 1% | 75 | 21152079 |
|  | Barrett's Esophagus | 6.48E-12 | 6.159 | 1% | 15 | 21152079 |
|  | Esophageal Adenocarcinoma | 7.39E-5 | 2.488 | 1% | 8 | 15833844 |
|  | Barrett's Esophagus | 2.37E-4 | 2.085 | 1% | 8 | 15833844 |
| Gastric cancer | Gastric Cancer | 2.06E-5 | 2.156 | 3% | 80 | 20965966 |
|  | Gastric Intestinal Type Adenocarcinoma | 6.18E-5 | 2.524 | 21% | 26 | 19081245 |
| Head and neck cancer | Head and Neck Squamous Cell Carcinoma | 1.27E-5 | 4.515 | 2% | 34 | 14676830 |
|  | Tongue Squamous Cell Carcinoma | 1.01E-4 | 2.205 | 15% | 31 | 19138406 |
| Kidney Cancer | Clear Cell Renal Cell Carcinoma | 1.42E-5 | -4.225 | 1% | 9 | 14641932 |
|  | Clear Cell Renal Cell Carcinoma | 2.81E-7 | -5.800 | 2% | 10 | 17699851 |
|  | Chromophobe Renal Cell Carcinoma | 1.03E-9 | -4.578 | 3% | 6 | 16115910 |
|  | Renal Oncocytoma | 6.67E-8 | -4.490 | 8% | 12 | 16115910 |
|  | Papillary Renal Cell Carcinoma | 1.15E-7 | -3.297 | 8% | 11 | 16115910 |
|  | Renal Pelvis Urothelial Carcinoma | 7.27E-4 | -3.798 | 16% | 8 | 16115910 |
|  | Non-Hereditary Clear Cell Renal Cell Carcinoma | 1.01E-4 | -3.964 | 10% | 27 | 19470766 |
|  | Hereditary Clear Cell Renal Cell Carcinoma | 2.31E-4 | -3.521 | 19% | 32 | 19470766 |
| Leukemia | Chronic Lymphocytic Leukemia | 1.03E-9 | 7.449 | 3% | 34 | 15778709 |
|  | Hairy Cell Leukemia | 1.36E-9 | 7.307 | 3% | 16 | 15778709 |
|  | Acute Myeloid Leukemia | 3.31E-7 | -8.468 | 1% | 9 | 14770183 |
|  | T-Cell Acute Lymphoblastic Leukemia | 2.70E-40 | -3.376 | 3% | 174 | 20406941 |
|  | B-Cell Childhood Acute Lymphoblastic Leukemia | 3.80E-40 | -3.247 | 3% | 359 | 20406941 |
|  | B-Cell Acute Lymphoblastic Leukemia | 6.56E-37 | -3.107 | 4% | 147 | 20406941 |
|  | Pro-B Acute Lymphoblastic Leukemia | 1.65E-29 | -2.752 | 4% | 70 | 20406941 |
|  | T-Cell Acute Lymphoblastic Leukemia | 1.07E-4 | -6.911 | 15% | 11 | 17410184 |
|  | B-Cell Acute Lymphoblastic Leukemia | 2.23E-4 | -3.991 | 21% | 87 | 17410184 |
| Lung cancer | Lung Carcinoid Tumor | 1.31E-13 | -98.420 | 1% | 20 | 11707567 |
|  | Lung Adenocarcinoma | 2.23E-6 | -6.251 | 3% | 132 | 11707567 |
|  | Squamous Cell Lung Carcinoma | 7.36E-5 | -6.324 | 4% | 21 | 11707567 |
|  | Small Cell Lung Carcinoma | 3.34E-5 | -9.271 | 5% | 6 | 11707567 |
|  | Lung Adenocarcinoma | 5.45E-22 | -3.116 | 3% | 58 | 22613842 |
|  | Squamous Cell Lung Carcinoma | 3.06E-4 | -4.644 | 7% | 13 | 11707590 |
|  | Large Cell Lung Carcinoma | 7.79E-8 | -5.036 | 9% | 19 | 20421987 |
|  | Squamous Cell Lung Carcinoma | 5.81E-9 | -2.601 | 13% | 27 | 20421987 |
| Lymphoma | Mantle Cell Lymphoma | 9.45E-10 | 9.760 | 1% | 8 | 15778709 |
|  | Centroblastic Lymphoma | 1.41E-7 | 4.891 | 9% | 28 | 15778709 |
|  | Burkitt's Lymphoma | 6.66E-4 | 2.627 | 17% | 17 | 15778709 |
|  | Unspecified Peripheral T-Cell Lymphoma | 1.54E-17 | 5.266 | 1% | 28 | 17304354 |
|  | Anaplastic Large Cell Lymphoma | 1.68E-4 | 9.110 | 12% | 6 | 17304354 |
|  | Angioimmunoblastic T-Cell Lymphoma | 1.86E-4 | 6.804 | 12% | 6 | 17304354 |
|  | Nodular Lymphocyte Predominant Hodgkin's Lymphoma | 1.85E-7 | 2.999 | 1% | 5 | 18794340 |
|  | Germinal Center B-Cell-Like Diffuse Large B-Cell Lymphoma | 8.40E-6 | 2.812 | 4% | 9 | 19412164 |
|  | Activated B-Cell-Like Diffuse Large B-Cell Lymphoma | 2.24E-9 | 3.772 | 5% | 17 | 19412164 |
|  | Diffuse Large B-Cell Lymphoma | 5.73E-15 | 3.586 | 5% | 44 | 19412164 |
|  | Follicular Lymphoma | 4.79E-11 | 2.569 | 7% | 38 | 19412164 |
|  | Anaplastic Large Cell Lymphoma, ALK-Positive | 9.49E-5 | 2.769 | 5% | 5 | 19657361 |
| Melanoma | Melanoma | 1.30E-4 | 2.505 | 3% | 6 | 15833814 |
| Pancreatic cancer | Pancreatic Adenocarcinoma | 4.28E-8 | 4.790 | 1% | 12 | 12651607 |
|  | Pancreatic Ductal Adenocarcinoma | 2.22E-5 | 2.121 | 23% | 39 | 19260470 |
| Sarcoma | Synovial Sarcoma | 4.15E-5 | -4.368 | 1% | 4 | 15994966 |
|  | Gastrointestinal Stromal Tumor | 3.81E-4 | -2.648 | 9% | 6 | 21447720 |
