# Supplementary figures and images for: SLC7A7 is a prognostic biomarker correlated with immune infiltrates in non-small cell lung cancer
Source: Cancer Cell Int. 2021 Feb 15;21:106. doi: 10.1186/s12935-021-01781-7 (PMC7905560; doi:10.1186/s12935-021-01781-7)

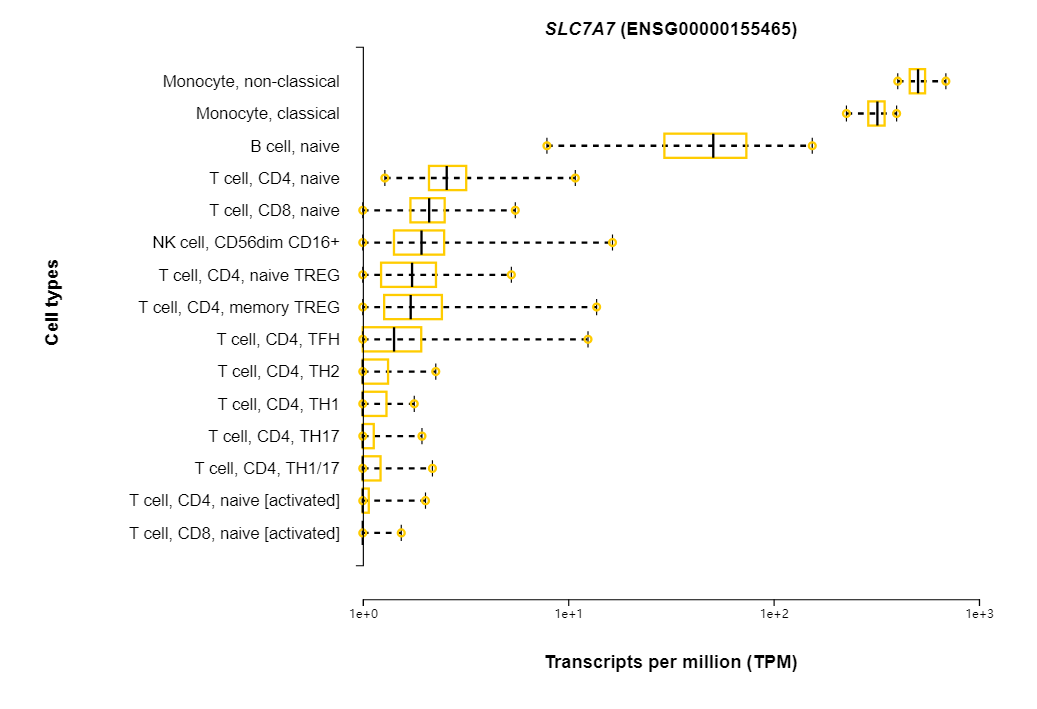

Supplement: Supplementary file 2 — Additional file 2. Boxplot of expression of SLC7A7 across diverse immune cells. [file 12935_2021_1781_MOESM2_ESM.png]

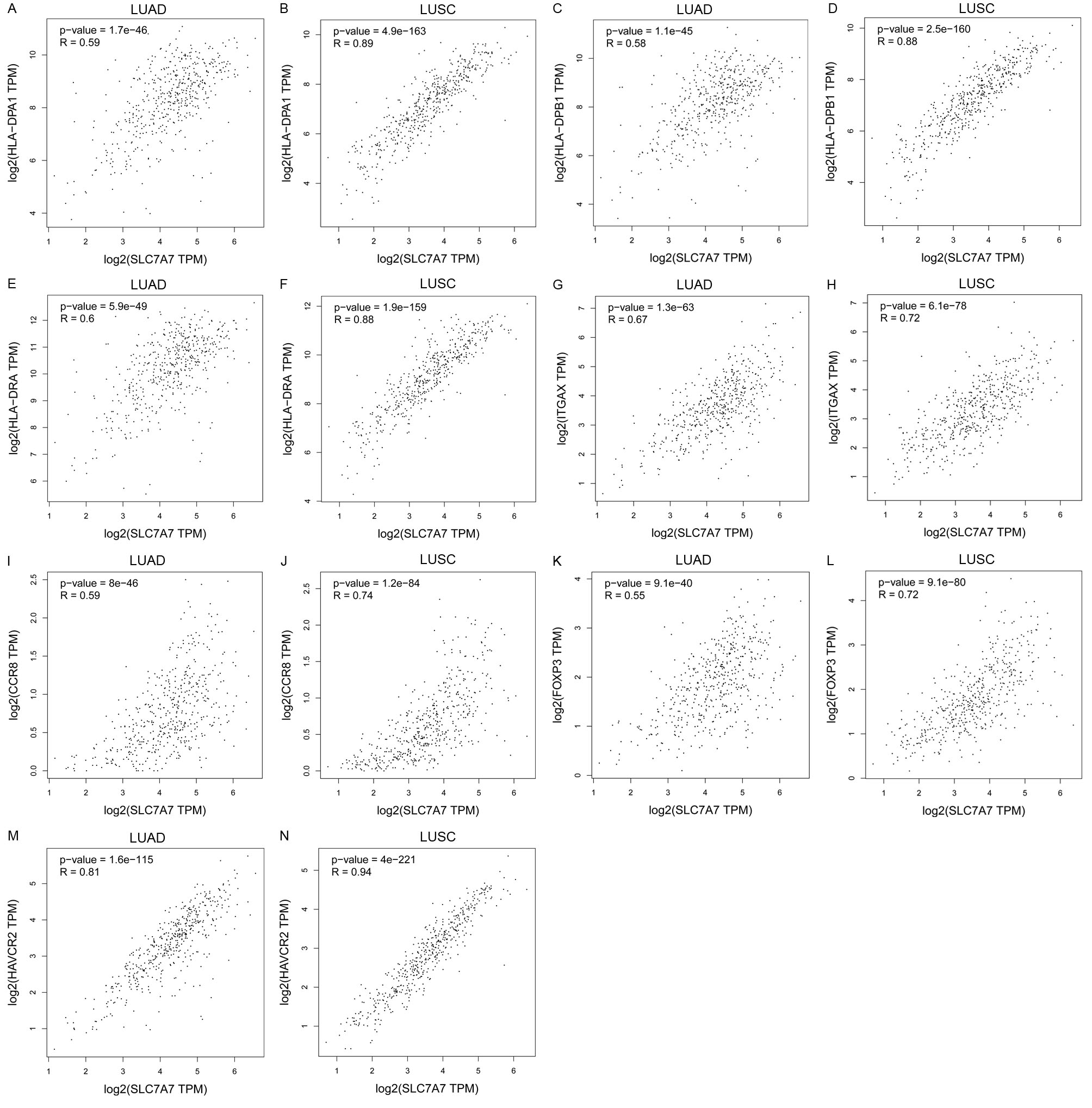

Supplement: Supplementary file 3 — Additional file 3. The correlations between SLC7A7 and makers of monocytes and TAMs were validated by GEPIA. [file 12935_2021_1781_MOESM3_ESM.jpg]
